# Supplementary material for: Genome-wide identification, characterization and gene expression of BES1 transcription factor family in grapevine (Vitis vinifera L.)
Source: Sci Rep. 2023 Jan 5;13:240. doi: 10.1038/s41598-022-24407-y (PMC9816167; doi:10.1038/s41598-022-24407-y)
Supplement: Supplementary file 3 — Supplementary Information. [file 41598_2022_24407_MOESM3_ESM.zip › Vvi_Atr/Vitis_vinifera.PN40024.v4.dna_sm.toplevel.fa.vs.Amborella_trichopoda.AMTR1.0.dna_sm.toplevel.fa.html/Atr-AmTr_v1.0_scaffold00161.html]

|  |  |  |  |  |  |  |  |  |  |  |  |  |  |
| --- | --- | --- | --- | --- | --- | --- | --- | --- | --- | --- | --- | --- | --- |
| Duplication depth | Reference chromosome | Collinear blocks | | | | | | | | | | | |
| 0 | Atr-ERN10469 |  |  |  |  |  |  |  |  |  |  |  |  |
| 0 | Atr-ERN10470 |  |  |  |  |  |  |  |  |  |  |  |  |
| 0 | Atr-ERN10471 |  |  |  |  |  |  |  |  |  |  |  |  |
| 0 | Atr-ERN10472 |  |  |  |  |  |  |  |  |  |  |  |  |
| 0 | Atr-ERN10473 |  |  |  |  |  |  |  |  |  |  |  |  |
| 0 | Atr-ERN10474 |  |  |  |  |  |  |  |  |  |  |  |  |
| 0 | Atr-ERN10475 |  |  |  |  |  |  |  |  |  |  |  |  |
| 0 | Atr-ERN10476 |  |  |  |  |  |  |  |  |  |  |  |  |
| 0 | Atr-ERN10477 |  |  |  |  |  |  |  |  |  |  |  |  |
| 0 | Atr-ERN10478 |  |  |  |  |  |  |  |  |  |  |  |  |
| 0 | Atr-ERN10479 |  |  |  |  |  |  |  |  |  |  |  |  |
| 0 | Atr-ERN10480 |  |  |  |  |  |  |  |  |  |  |  |  |
| 0 | Atr-ERN10481 |  |  |  |  |  |  |  |  |  |  |  |  |
| 0 | Atr-ERN10482 |  |  |  |  |  |  |  |  |  |  |  |  |
| 0 | Atr-ERN10483 |  |  |  |  |  |  |  |  |  |  |  |  |
| 0 | Atr-ERN10484 |  |  |  |  |  |  |  |  |  |  |  |  |
| 0 | Atr-ERN10485 |  |  |  |  |  |  |  |  |  |  |  |  |
| 0 | Atr-ERN10486 |  |  |  |  |  |  |  |  |  |  |  |  |
| 0 | Atr-ERN10487 |  |  |  |  |  |  |  |  |  |  |  |  |
| 0 | Atr-ERN10488 |  |  |  |  |  |  |  |  |  |  |  |  |
| 0 | Atr-ERN10489 |  |  |  |  |  |  |  |  |  |  |  |  |
| 0 | Atr-ERN10490 |  |  |  |  |  |  |  |  |  |  |  |  |
| 0 | Atr-ERN10491 |  |  |  |  |  |  |  |  |  |  |  |  |
| 0 | Atr-ERN10492 |  |  |  |  |  |  |  |  |  |  |  |  |
| 0 | Atr-ERN10493 |  |  |  |  |  |  |  |  |  |  |  |  |
| 0 | Atr-ERN10494 |  |  |  |  |  |  |  |  |  |  |  |  |
| 0 | Atr-ERN10495 |  |  |  |  |  |  |  |  |  |  |  |  |
| 0 | Atr-ERN10496 |  |  |  |  |  |  |  |  |  |  |  |  |
| 0 | Atr-ERN10497 |  |  |  |  |  |  |  |  |  |  |  |  |
| 0 | Atr-ERN10498 |  |  |  |  |  |  |  |  |  |  |  |  |
| 0 | Atr-ERN10499 |  |  |  |  |  |  |  |  |  |  |  |  |
| 0 | Atr-ERN10500 |  |  |  |  |  |  |  |  |  |  |  |  |
| 0 | Atr-ERN10501 |  |  |  |  |  |  |  |  |  |  |  |  |
